# LoCS-Net: Localizing convolutional spiking neural network for fast visual place recognition

**DOI:** 10.3389/fnbot.2024.1490267

**Published:** 2025-01-29

**Authors:** Ugur Akcal, Ivan Georgiev Raikov, Ekaterina Dmitrievna Gribkova, Anwesa Choudhuri, Seung Hyun Kim, Mattia Gazzola, Rhanor Gillette, Ivan Soltesz, Girish Chowdhary

**Affiliations:** ^1^The Grainger College of Engineering, Department of Aerospace Engineering, University of Illinois Urbana-Champaign, Urbana, IL, United States; ^2^The Grainger College of Engineering, Siebel School of Computing and Data Science, University of Illinois Urbana-Champaign, Urbana, IL, United States; ^3^Coordinated Science Laboratory, University of Illinois Urbana-Champaign, Urbana, IL, United States; ^4^Department of Neurosurgery, Stanford University, Stanford, CA, United States; ^5^Neuroscience Program, Center for Artificial Intelligence Innovation, University of Illinois Urbana-Champaign, Urbana, IL, United States; ^6^The Grainger College of Engineering, Department of Electrical and Computer Engineering, University of Illinois Urbana-Champaign, Urbana, IL, United States; ^7^The Grainger College of Engineering, Mechanical Science and Engineering, University of Illinois Urbana-Champaign, Urbana, IL, United States; ^8^Department of Molecular and Integrative Physiology, University of Illinois Urbana-Champaign, Urbana, IL, United States; ^9^The Grainger College of Engineering, College of Agriculture and Consumer Economics, Department of Agricultural and Biological Engineering, University of Illinois Urbana-Champaign, Urbana, IL, United States

**Keywords:** spiking neural networks, robotics, visual place recognition, localization, supervised learning, convolutional networks

## Abstract

Visual place recognition (VPR) is the ability to recognize locations in a physical environment based only on visual inputs. It is a challenging task due to perceptual aliasing, viewpoint and appearance variations and complexity of dynamic scenes. Despite promising demonstrations, many state-of-the-art (SOTA) VPR approaches based on artificial neural networks (ANNs) suffer from computational inefficiency. However, spiking neural networks (SNNs) implemented on neuromorphic hardware are reported to have remarkable potential for more efficient solutions computationally. Still, training SOTA SNNs for VPR is often intractable on large and diverse datasets, and they typically demonstrate poor real-time operation performance. To address these shortcomings, we developed an end-to-end convolutional SNN model for VPR that leverages backpropagation for tractable training. Rate-based approximations of leaky integrate-and-fire (LIF) neurons are employed during training, which are then replaced with spiking LIF neurons during inference. The proposed method significantly outperforms existing SOTA SNNs on challenging datasets like Nordland and Oxford RobotCar, achieving 78.6% precision at 100% recall on the Nordland dataset (compared to 73.0% from the current SOTA) and 45.7% on the Oxford RobotCar dataset (compared to 20.2% from the current SOTA). Our approach offers a simpler training pipeline while yielding significant improvements in both training and inference times compared to SOTA SNNs for VPR. Hardware-in-the-loop tests using Intel's neuromorphic USB form factor, Kapoho Bay, show that our on-chip spiking models for VPR trained via the ANN-to-SNN conversion strategy continue to outperform their SNN counterparts, despite a slight but noticeable decrease in performance when transitioning from off-chip to on-chip, while offering significant energy efficiency. The results highlight the outstanding rapid prototyping and real-world deployment capabilities of this approach, showing it to be a substantial step toward more prevalent SNN-based real-world robotics solutions.

## 1 Introduction

Visual place recognition (VPR) refers to the capability of identifying locations within a physical environment solely through visual inputs. It is essential for autonomous navigation of mobile robots, indoor assistive navigation aid, augmented reality, and geolocalization (Lanham, 2018; Reinhardt, 2019; Weyand et al., 2016; Seo et al., 2018; Li et al., 2018; Shan et al., 2015). These applications generally involve complex dynamic scenes, perceptual aliasing, viewpoint and appearance variation, which render VPR extremely challenging.

VPR has been approached via deep learning techniques (Radenović et al., 2018; Chen et al., 2017; Sünderhauf et al., 2015) and through various supervised and self-supervised feature descriptor representations (DeTone et al., 2018; He et al., 2018; McManus et al., 2014). Despite their promise, many of these methods face significant practical challenges (Lynen et al., 2015, 2020). For example, they often rely on large, deep networks with time-consuming training processes and dense feature extraction, ultimately making them computationally expensive, memory-intensive, and energy-demanding. Such limitations significantly reduce the ability for real-world deployment of conventional artificial neural networks (ANNs) on robotic platforms with limited on-board resources (Doan et al., 2019). Spiking neural networks (SNNs) offer an alternative with their remarkable potential for computationally efficient operation when they are implemented on neuromorphic hardware (Davies et al., 2021). However, previous work on SNN models for VPR has suffered from scalability problems that impede their application to data with a large number of locations. In addition, the majority of the aforementioned methods formulate VPR as an image retrieval task (Garg et al., 2021), the solution of which aims for the correct association of given query images with a set of reference images. Such formulation requires the employment of a confusion matrix (a.k.a. distance matrix) (Garg et al., 2022) populated with similarity scores based on the distances between model-specific feature descriptors. A commonly-used similarity metric is the cosine similarity (Naseer et al., 2018), which is reported to be computationally expensive when evaluating high-dimensional feature vectors (Zhang et al., 2021).

These drawbacks have motivated our approach to VPR, described in this paper, in which an SNN model is implemented using an ANN-to-SNN conversion method to enable backpropagation-based training, resulting in fast training and inference times. We employ a smooth rate-based approximation (Hunsberger and Eliasmith, 2015) of the leaky integrate-and-fire (LIF) neurons (Burkitt, 2006) during the training. Once the training session is completed the rate-based units are substituted with the spiking LIF neurons and the resulting spiking network is used for inference.

We formulate VPR as a classification task, where the SNN model predicts place labels that uniquely correspond to the locations in a discretized navigation domain. We evaluate our method with the challenging real-world benchmark datasets Nordland (Olid et al., 2018) and Oxford RobotCar (Maddern et al., 2017, 2020). Our model, the Localizing Convolutional Spiking Neural Network (LoCS-Net), outperforms other SOTA SNN-based VPR methods on both the Nordland (Olid et al., 2018) and the Oxford RobotCar dataset (Maddern et al., 2017, 2020) in terms of precison at 100% recall (P@100%R).

The main contributions of this work are as follows. (a) To the best of our knowledge, LoCS-Net is the first SNN that is trained to perform the VPR task by means of ANN-to-SNN conversion and backpropagation. (b) LoCS-Net is an end-to-end SNN solution. Therefore, LoCS-Net does not require further processing of its outputs for recognizing places. In that sense, LoCS-Net saves all the computation resources that traditional VPR algorithms would typically expend on feature encoding, descriptor matching, computing similarity scores, and storing a distance matrix. (c) We demonstrate that our proposed SNN model yields the fastest training time, the second fastest inference time, and the best VPR performance in P@100%R among its SNN counterparts. This poses LoCS-Net as a significant step toward deployment of SNN-based VPR systems on robotics platforms for real-time localization. (d) We report the challenges we experienced when deploying LoCS-Net on the neuromorphic Loihi chips in detail. We strongly believe that our in-depth discussion on hardware deployment will be useful for the SNN-VPR community.

## 2 Related work

Task-specific feature descriptors are the very core of traditional VPR systems, which can be grouped into two categories: (1) Local descriptors, (2) Global descriptors. Local descriptors may scan the given images in patches of arbitrary size and stride. These patches are then compared to their immediate neighborhood to determine the distinguishing patterns (Loncomilla et al., 2016). In general, previous VPR work utilizing local descriptors (Johns and Yang, 2011; Kim et al., 2015; Zemene et al., 2018) employs sparse filters that extract so-called key-points (Mikolajczyk and Schmid, 2002; Matas et al., 2004). These key-points can be marked by the descriptions generated through the application of methods including SIFT (Lowe, 1999), RootSIFT (Arandjelović and Zisserman, 2012), SURF (Bay et al., 2006), and BRIEF (Calonder et al., 2011). In this way, the combination of heuristics-based detectors and local descriptors can be used for: (A) Representing images, (B) Comparing two images with respect to their descriptors to determine how similar they are. In addition, local features can be combined with other embeddings (Tsintotas et al., 2022) while leveraging their robustness against the variations in the robot's pose. However, local descriptors can be computationally heavier and more sensitive to illumination changes (Masone and Caputo, 2021). Global descriptors (Oliva and Torralba, 2006; Torralba et al., 2008), on the other hand, do not require a detection phase and directly encode the holistic properties of the input images. Although this might save the global descriptor-based VPR methods (Liu and Zhang, 2012; Schönberger et al., 2018; Revaud et al., 2019; Yin et al., 2019) some compute time, they are more vulnerable to robot pose changes than their local descriptor-based counterparts while being inept at capturing geometric structures (Dube et al., 2020). Yet, global descriptors are reported to be more effective in the case of varying lighting conditions (Lowry et al., 2015). Furthermore, there are hybrid approaches (Siméoni et al., 2019; Cao et al., 2020; Hausler et al., 2021), which combine the strengths of both approaches.

Deep learning has made key contributions to recent work on VPR. An influential deep-learning-based approach is NetVLAD (Arandjelovic et al., 2016), which is a supervised method for place recognition, based on the Vector of Locally Aggregated Descriptors (VLAD), a technique to construct global image feature representations from local feature descriptors. NetVLAD uses a pre-trained feature extraction network, such as AlexNet (Krizhevsky et al., 2017), to extract the local features, and a loss function that aims to minimize the distance between a baseline input and the most similar image (the positive example), while maximizing the distance between baseline input and the most dissimilar image (the negative example). This loss function is also known as the triplet loss function. Several authors have extended NetVLAD in different directions, and NetVLAD-based methods still perform very competitively (Hausler et al., 2021; Yu et al., 2020).

SNNs have been of interest for various robotics tasks, including not only VPR, but also object detection (Kim et al., 2020), regression (Gehrig et al., 2020), and control of aerial platforms (Vitale et al., 2021) due to their significant potential for computational efficiency (Zhu et al., 2020). Published VPR methods based on SNNs are relatively recent, compared to other robotics research areas. Among them, Hussaini et al. (2022) is reported to be the first high-performance SNN for VPR. There, the authors propose a feed-forward SNN, where the output neuron activations are filtered through a custom softmax layer. Follow-up work by the same authors (Hussaini et al., 2023) introduced a framework where localized spiking neural ensembles are trained to recognize places in particular regions of the environment. They further regularize these networks by removing output from “hyper-active neurons,” which exhibit intense spiking activity when provided with input from the regions outside of the ensemble's expertise. This framework yields a significant improvement over its predecessor while demonstrating either superior or competitive VPR performance compared to the traditional methods. A recent study by Hines et al. (2024) presented an SNN model composed of an ensemble of modified BliTNet (Stratton et al., 2022) modules, each tuned to specific regions within the navigation domain. During training, spike forcing is utilized to encode locations uniquely, which are later identified by monitoring the output neuron with the highest spike amplitude. The authors report remarkable improvements in both training and inference times, alongside achieving superior or comparable VPR performance compared to earlier SNN models. However, training of these SNN approaches do not scale with the increasing volume of training data. In addition, heuristics such as the assignment of neural ensembles to spatial regions, nearest neighbor search in the similarity matrix, and the regularization process further complicate the training process and the computational efficiency of the model. In contrast to these previous SNN-based approaches, we propose an end-to-end solution that is much easier to train and to deploy without requiring heuristic training.

## 3 LoCS-Net model for visual place recognition

Here, we begin with an overview of the task formulation and the architecture of LoCS-Net in Section 3.1. Section 3.2 formally poses the VPR problem as a classification task. Then, in Section 3.3, we walk through the LoCS-Net pipeline and its key design choices. Moreover, Section 3.3 provides a summary of the ANN-to-SNN conversion paradigm while elaborating on its use for the present work. We would like to refer the readers to the supplementary information and to the figshare repository of our code for further implementation details: https://figshare.com/s/c159a8680a261ced28b2.

### 3.1 Overview

**Figure 1:**
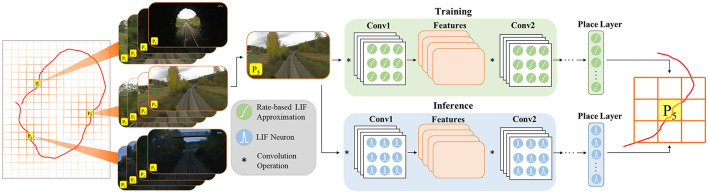
LoCS-Net VPR system: a convolutional network of rate-based LIF neurons (Hunsberger and Eliasmith, 2015) is trained over a set of annotated images sampled over a trajectory (the red curve) traversing a finite discretized (orange grid bounded by gray lines) navigation domain. The VPR task is formulated as a classification problem where each tile of the grid (*P*_1_, *P*_2_, *P*_3_, …) corresponds to a distinct location. After training, the LIF approximations are substituted with the spiking LIF neurons (Burkitt, 2006) for the inference step.

Figure 1 depicts the overall architecture of LoCS-Net. The input to the model is a set of images sampled along a trajectory that traverses a bounded navigation domain. The domain is discretized by means of a uniform grid (orange lines in Figure 1) and each image is assigned an integer *place* label based on the tile traversed at the time of sampling the image. In this manner, we define the VPR task as a classification problem as discussed in Section 3.2.

Each layer in the LoCS-Net model consists of LIF neurons (Burkitt, 2006). In order to train the model, these neurons are converted to rate-based approximations of LIF units (Hunsberger and Eliasmith, 2015). Rate-based LIF approximations are continuous differentiable representations of the LIF activation function. The LIF activation function describes the time evolution of the neuron's membrane potential, and it is discontinuous: when the membrane potential reaches a threshold value, it is reset back to a pre-determined state. The rate-based approximation is a continuous function that describes the neuron's firing rate as a function of its input, enabling the use of back-propagation algorithms for training. However, this doesn't prevent the substitution of the approximate LIF neurons with the original ones for inference after the training is complete. A number of authors have reported successful applications (Rueckauer et al., 2017; Hu et al., 2021; Patel et al., 2019) of ANN-to-SNN conversion.

### 3.2 VPR as a classification task

A common practice in approaching the VPR task is to pose it as an image retrieval problem where the goal is to compute and store descriptors that would effectively encode both the set of query images and the collection of reference images to match (Lajoie and Beltrame, 2022). The encoding process is followed by an image retrieval scheme, which is based on comparing query embeddings (z_q_) to the database of reference descriptors (z_r_) with respect to the customized similarity metrics. Nevertheless, computation of the descriptors is numerically expensive. In contrast, we formulate the VPR task as a classification problem in order to bypass the encoding phase of the images. We designed the LoCS-Net so that it would uniquely map the given input images to the mutually exclusive classes, which are the distinct places, as discussed in Sections 3.1, 3.3.

**Figure 2:**
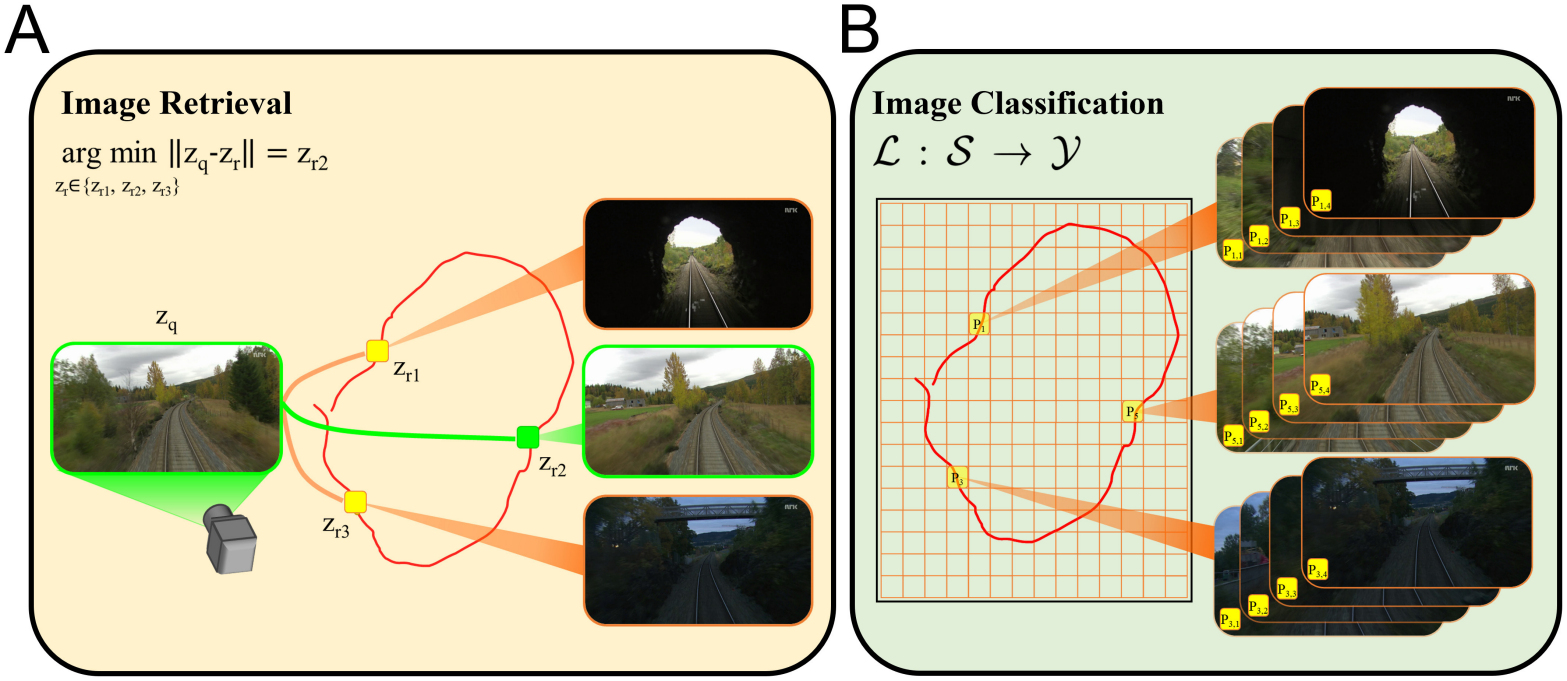
VPR can be posed as image retrieval task or image classification problem. For both formulations we consider a set of images collected over a trajectory (the red curves) traversing a finite navigation domain. **(A)** A popular VPR solution is based on generating descriptors for query (z_q_) and reference images (z_r_), which are then compared to each other in terms of a distance (or similarity) metric ∥·∥ in order to retrieve the reference image corresponding to the correct place. For instance, z_r2_ represents the most similar reference image to the given query image. **(B)** In contrast, the image classification formulation of the VPR task requires an arbitrary discretization of the navigation domain to define the classes *P*_*i*_ (the places where i∈Y) that annotate the images s∈S. Then, a classifier L is trained to map images s∈S to the correct place labels i∈Y. The image annotation *P*_*i, j*_ denotes the *j*^th^ image associated with the class *P*_*i*_.

Figure 2 illustrates how our work formulates VPR differently compared to the image retrieval VPR formulation. We first discretize the navigation domain by using a uniform rectangular grid (Figure 2B, the orange lines). Here, each tile of the grid defines a distinct place *P*_*i*_, *i* = 1, 2, 3, ⋯ . We would like to note that the navigation domain can be any physical environment with points described by spatial coordinates. Although we use a uniform rectangular grid to discretize the top-down view of the domain of interest, our approach is flexible with respect to the definition of places, and permits 3-D as well as 2-D discretization. As one of many ways to generate the training and test data, we sample images over numerous trajectories traversing the discretized navigation domain. Suppose that an image s∈S is sampled at the time instant when the camera is in the region represented by tile *P*_5_. Then, this image would be annotated by the place label 5∈Y. Namely, the image *s* belongs to the class represented by the tile *P*_5_. Thus, given a query image, our goal is to train a spiking neural network model that would correctly infer the associated place labels. Hence, we pose the VPR task as an image classification problem in this fashion. We now formally describe the VPR task as a classification problem as follows.

Consider a set of images, $S=\left\{s\in {\mathbb{R}}^{C\times H\times W}|X\left(s\right)\in D\right\}$, where *C* is the number of color channels, *H* and *W* are the height and width of the images in pixels and D is a pre-determined finite horizontal navigation domain. Here, X:S→D is a function that maps the images s∈S to the planar spatial coordinates ${\left[x_{s},y_{s}\right]}^{\text{T}}\in D=\left\{{\left[d_{1},d_{2}\right]}^{\text{T}}\in {\mathbb{R}}^{2}|x_{\text{min}}\leq d_{1}\leq x_{\text{max}}\wedge y_{\text{min}}\leq d_{2}\leq y_{\text{max}}\right\}$ where *x*_min_, *x*_max_, *y*_min_, and *y*_max_ are the bounds of D. *X*(*s*) describes the in-plane spatial state of the camera with respect to a local frame of choice when s∈S is sampled. The set $Y=\left\{i\in \mathbb{N}|i\leq N_{P}\right\}$ contains the place labels that annotate s∈S where *N*_*P*_ is the number of assumed places. Each y∈Y corresponds to a $P_{y}\subset D$ such that $P_{y}\cap P_{i}\equiv \emptyset ,y\neq i\wedge i\in Y$. We formulate the VPR task as an image classification problem, where each class is assumed to be mutually exclusive. That is, each image belongs exactly to one class. Our goal is to design a mapping L:S→Y that correctly predicts the place label y∈Y of any given s∈S. One should note that the approach we describe here is different than the image retrieval formulation as we want L to predict the place labels instead of directly associating the input images with the reference images.

### 3.3 Localizing convolutional spiking neural network

The design of LoCS-Net is defined mainly by two ideas: (1) Discretization of the given finite navigation domain, (2) Leveraging the back-propagation algorithm by adopting the ANN-to-SNN conversion paradigm. We now walk through the details of these ideas together with the architecture of LoCS-Net and its building blocks, LIF neurons.

#### 3.3.1 The LIF neuron model

Unlike standard artificial neurons, which are defined by time-independent differentiable non-linear transfer functions with continuous outputs, spiking neurons have time-dependent dynamics that aim to capture the information processing in the biological neural systems by emitting discrete pulses (Burkitt, 2006). Equation 1 describes the dynamics of an LIF neuron.


(1) $$\begin{array}{l} C_{m}\frac{d\nu \left(t\right)}{dt}=-\frac{C_{m}}{\tau_{m}}\left[\nu \left(t\right)-\nu_{0}\right]+I_{s}\left(t\right)+I_{inj}\left(t\right) \end{array}$$

where *C*_*m*_ is the membrane capacitance, τ_*m*_ is the passive membrane time constant, and ν_0_ is the resting potential. Above formulation considers a resetting scalar state variable, the membrane potential ν(*t*), which will be reinitialized at ν(*t*) = ν_*reset*_ after reaching a threshold, ν(*t*) = ν_*th*_. Whenever the re-initialization happens at time *t* = *t*_*spike*_, the output of the LIF neuron (*o*(*t*)) will be an impulse signal of unity. We name this a spike event. One can express a spike event of an LIF neuron by Equation 2, which incorporates Dirac's delta function centered at the time of re-initialization.


(2) $$\begin{array}{l} o\left(t_{spike}\right)=\delta \left[\nu \left(t_{spike}\right)-\nu_{th}\right] \end{array}$$

The right hand side of Equation 1 includes three terms: (1) An exponential decay term (a.k.a the passive membrane leak), (2) *I*_*s*_(*t*), the sum of incoming synaptic currents, which are mostly unit impulses filtered through a first order delay and/or multiplied by some scalar, and finally (3) An injection term, *I*_*inj*_(*t*), that describes the input currents other than synaptic currents. This can be some bias representing the background noise in the corresponding neural system, or just some external input.

Solving the sub-threshold dynamics described by Equation 1 for the firing rate ρ[*I*_*s*_(*t*)] of an LIF neuron and assuming *I*_*inj*_(*t*) = 0 for all *t*≥0 yields the following.


(3) $$\begin{array}{l} T_{spike}=-\tau_{m}\log\left(1-\frac{\left(\nu_{th}-\nu_{reset}\right)\frac{C_{m}}{\tau_{m}}}{\left(\nu_{0}-\nu_{reset}\right)\frac{C_{m}}{\tau_{m}}+I_{s}\left(t\right)}\right) \end{array}$$

(4) $$\rho [I_{s}(t)]=\{\begin{array}{cc} 0 & \text{if }I_{s}(t)\leq I_{th}\\ \frac{1}{T_{ref}+T_{spike}} & \text{if }I_{s}(t)>I_{th} \end{array};I_{th}=(\nu_{th}-\nu_{0})\frac{C_{m}}{\tau_{m}}$$

*T*_*ref*_ is the refractory period, which is the time it takes a neuron to start accepting input currents after a spike event. *T*_*spike*_ is the time it takes a neuron to reach ν_*th*_ from ν_*reset*_ after a spike event at some *t* = *t*′ given $\nu_{th}<I_{s}\left(t\right)=c\in \mathbb{R},t^{\prime }<t\leq t^{\prime }+T_{spike}$. Equations 3, 4 describe the response curve of an LIF neuron, which has a discontinuous and unbounded derivative (∂ρ/∂*I*_*s*_) at *I*_*s*_ = (ν_*th*_−ν_0_)*C*_*m*_/τ_*m*_. However, one can modify (Equation 4) as described by Hunsberger and Eliasmith (2015) in order to obtain a smooth rate-based LIF approximation.


(5) $$\begin{array}{lll} \rho^{\prime }\left[I_{s}\left(t\right)\right] & = & {\left\{T_{ref}+\tau_{m}\log\left(1+\frac{\nu_{th}}{\Theta \left[I_{s}\left(t\right)-\nu_{th}\right]}\right)\right\}}^{-1};\\ \;\Theta \left(x\right) & = & \gamma \log\left(1+e^{x/\gamma }\right) \end{array}$$

where γ is the smoothing factor of choice.

#### 3.3.2 ANN-to-SNN conversion

**Figure 3:**
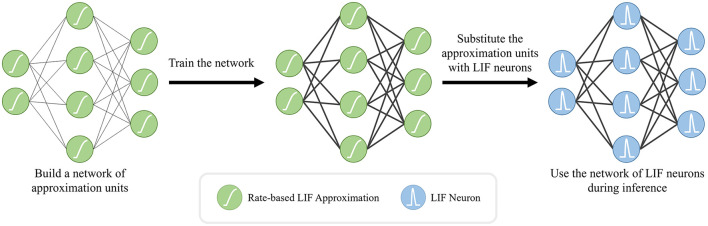
ANN-to-SNN conversion work-flow: we first employ rate-based approximations of the LIF neurons to train our network, since the discontinuous spike event outputs of the original LIF neurons prevents the training of the network through the back-propagation algorithm. After completing the training of this interim network, we substitute the LIF approximations with the original ones while keeping the network topology and the trained weights (bold black lines) the same.

Due to the discontinuities introduced by discrete spike events, the conventional gradient-descent training techniques need to be modified for spiking neural networks. Various approximation methods have been developed to overcome these discontinuities (Neftci et al., 2019). One such method is based on the utilization of the rate-based approximations, a.k.a. the tuning curves. Given a loss function, the main idea is to build a network of differentiable rate-based approximation units and solve for the synaptic weights by using an arbitrary version of gradient descent. Once the solution is obtained, the approximation units can be substituted with LIF neurons to use the resulting spiking network during inference as shown in Figure 3. We utilized NengoDL Rasmussen (2018) to implement the aforementioned ANN to SNN conversion methodology. We employed the standard sparse categorical cross entropy as our loss function.

#### 3.3.3 LoCS-Net architecture

**Figure 4:**
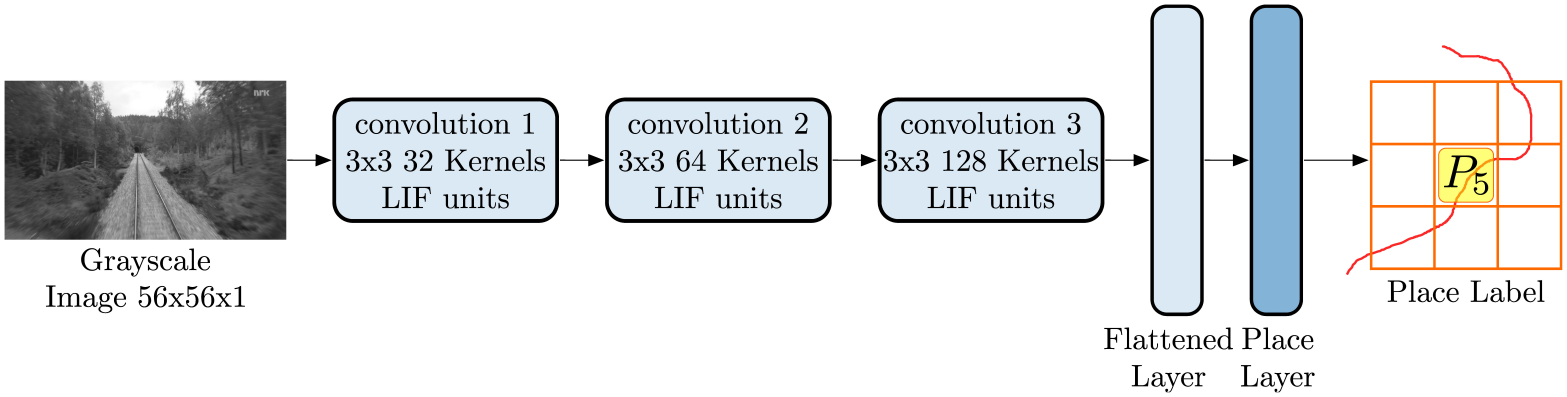
The LoCS-Net architecture consists of 3 convolutional layers followed by a fully connected output layer, known as the *place layer*. The units within this layer correspond to unique locations within the environment. LoCS-Net accepts 56 × 56 pixel grayscale images as inputs, using them to predict the associated places from which the input images were sampled.

As depicted in Figure 4, LoCS-Net is composed of a sequence of 3 convolutional layers followed by a fully connected output layer, also known as the “place layer,” the units of which correspond to distinct places in the environment. Inputs to LoCS-Net are grayscale images of 56 × 56 pixels. The number of neurons in the place layer is set to be the number of possible places (*N*_*P*_) as explained in Section 3.2. We considered 50 × 50 grid for Oxford RobotCar (ORC) data in our principal experiments. Note that for training, we employ the smooth rate-based approximated LIF units while maintaining the same architecture illustrated in Figure 4. We use sparse categorical cross entropy as the loss function during training. For inference, we replace the approximated LIF units of the trained network with spiking LIF neurons, keeping both the weights and the architecture unchanged. For further details of the network structure and the corresponding hyper-parameters, we refer the readers to the supplementary information and to the repository of the current work's code at https://figshare.com/s/c159a8680a261ced28b2.

## 4 Experiments

### 4.1 Datasets and evaluation metrics

We evaluate our proposed approach on the challenging Nordland (Olid et al., 2018) and ORC data (Maddern et al., 2017, 2020) following prior work (Hussaini et al., 2023). For the Nordland data experiments, we trained LoCS-Net using the spring and fall traverses and tested it with the summer traverse. For the ORC data experiments, we trained LoCS-Net on the sun (2015-08-12-15-04-18) and rain (2015-10-29-12-18-17) traverses, and tested its performance on the dusk (2014-11-21-16-07-03) traverse. We followed the Nordland data processing directions in Hussaini et al. (2023) for the same training and test data. We obtained 3,072 Nordland data (Olid et al., 2018) places, and 2,500 ORC data (Maddern et al., 2017, 2020) places (set by our grid definition) while considering the complete sun, rain, and dusk traverses used in Hussaini et al. (2023).

Although our discretization of the ORC domain yields a total of 2,500 possible places, the trajectories traversed in that dataset cover a much smaller number of labels. Some of the ORC data places are either occasionally visited or not visited at all. This is because the trajectories were generated by a vehicle traversing the road network, making it impossible to visit all parts of the spatial domain. Therefore, we filter out places that do not contain a minimum number (10) of unique training images. We also bound the number of unique instances per place from above (maximum 700) as the training of the baseline SNN models are getting infeasible due to increasing size of the data. Table 1 provides the training and the test data specifications yielded by our data pre-processing pipeline. We would like to note that LoCS-Net can still be trained and be tested on the full ORC data in a matter of minutes.

**Table 1:** LoCS-Net training and test data specifications.

**Specifications \dataset**	**Nordland**	**Oxford RobotCar (ORC)**
Train size [# of images]	6,144	32,475
Test size [# of images]	3,072	17,055
# of labels	3,072	2,500
# of unique labels	3,072	185

We employ standard VPR performance metrics, including the precision-recall curves, area-under-the-precision-recall curves (AUC-PR or AUC) (Cieslewski and Scaramuzza, 2017; Camara and Přeučil, 2019), and recall-at-N (R@N) curves (Perronnin et al., 2010; Uy and Lee, 2018) in order to assess the performance of our model.

### 4.2 Experimental set-up

**Figure 5:**
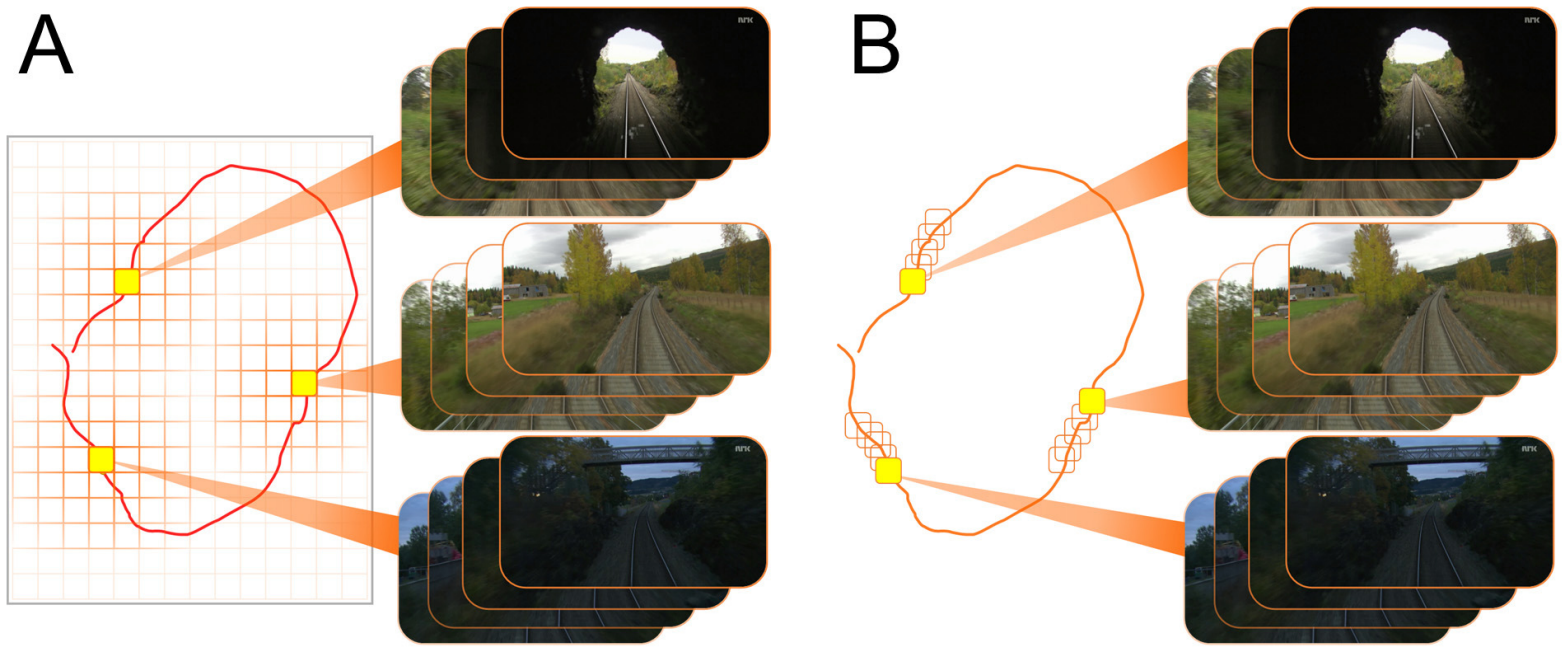
Annotating images: **(A)** top-down navigation domain is discretized by defining a grid of arbitrary resolution. Each tile of of the grid annotates the images sampled within its boundaries. **(B)** Images are sampled over a traverse at a pre-determined frequency while each image is corresponding to a unique place. For instance, if the first image is sampled at time *t* = 0*s*, then the second and the third image will be sampled at *t* = *Ts* and *t* = 2*Ts*, respectively.

We adopt two annotation methods as the Nordland (Olid et al., 2018) and the ORC data (Maddern et al., 2017, 2020) were structured in different ways. Figure 5A describes the labeling process of the ORC images (Maddern et al., 2017, 2020). As it is shown, we first encapsulated the top-down projection of the path within a rectangular region. Then, we discretize this region to obtain grid tiles, each of which represents a distinct place. These tiles annotate the images sampled within its boundaries.

To label the Nordland images (Olid et al., 2018) we followed the annotation method defined in Hussaini et al. (2023). As depicted in Figure 5B, we sample images over a traverse at a pre-determined frequency (every 8th image) while each image is corresponding to a unique place.

### 4.3 Quantitative results

We conducted several performance comparisons of LoCS-Net with the current SOTA SNN methods, Ensemble SNNs (Hussaini et al., 2023), VPRTempo (Hines et al., 2024), and Weighted Assignment SNN (WNA) (Hussaini et al., 2022). In order to save computational resources, we did not train and test WNA ourselves. Instead, in Table 2, we listed the performance metrics published in Table 1 of Hussaini et al. (2023). We also included additional performance comparisons of LoCS-Net to a set of ANN-based SOTA VPR techniques such as AP-GeM (Revaud et al., 2019), NetVLAD (Arandjelovic et al., 2016), MixVPR (Ali-Bey et al., 2023), Conv-AP (Ali-bey et al., 2022), EigenPlaces (Berton et al., 2023), and CosPlace (Berton et al., 2022). We utilized the benchmark tool developed by Berton et al. (2023) in order to perform these additional comparisons.

**Table 2:** VPR performance comparison in terms Precision at 100% Recall (P@100%R), area-under-the-precision-recall curves (AUC), mean inference time (MIT), mean training time (MIT), and effective energy consumed per inference.

		**Nordland**					**ORC**				
**Method**	**Approach**	**P@100%R**	**AUC**	**MIT [ms]**	**MTT [min]**	**Effective energy per inference [J]**	**P@100%R**	**AUC**	**MIT [ms]**	**MTT [min]**	**Effective energy per inference [J]**
LoCS-Net on GPU (ours)	SNN	**78.6%**	**0.980**	25	**1**	2.545	**45.7%**	**0.702**	10	**3.5**	1.095
LoCS-Net on NUC (ours)	SNN	**78.6%**	**0.980**	796	-	13.183	**45.7%**	**0.702**	371	-	5.871
LoCS-Net on Loihi (ours)	SNN	71.1%	0.761	288	-	**0.060**	41.0%	0.653	147	-	**0.032**
VPRTempo on GPU (Hines et al., 2024)	SNN	73.0%	0.975	**8**	15	0.079	20.2%	0.435	**5**	54	0.053
Ensemble SNNs on CPU (Hussaini et al., 2023)	SNN	66.9%	0.975	408	725	3.405	17.6%	0.485	290	3,408	3.051
WNA (Hussaini et al., 2022)	SNN	0.3%	0.005	-	-	-	4.0%	0.042	-	-	-
MixVPR (Ali-Bey et al., 2023)	ANN	**94.6%**	-	29	3	0.907	**87.7%**	-	14	**6**	0.578
Conv-AP (Ali-bey et al., 2022)	ANN	91.3%	-	27	**2**	0.847	84.6	-	18	8	0.632
EigenPlaces (Berton et al., 2023)	ANN	80.2%	-	57	5	6.443	71.5%	-	31	17	3.335
CosPlace (Berton et al., 2022)	ANN	75.3%	-	60	6	6.842	71.0%	-	35	17	3.524
AP-GeM (Revaud et al., 2019)	ANN	65.1%	-	95	9	10.512	60.7%	-	54	27	5.376
NetVLAD (Arandjelovic et al., 2016)	ANN	51.4%	-	107	10	12.641	43.8%	-	62	29	5.496

Bold values indicate the best performance metrics for SNN- and ANN-based approaches on individual datasets.

**Figure 6:**
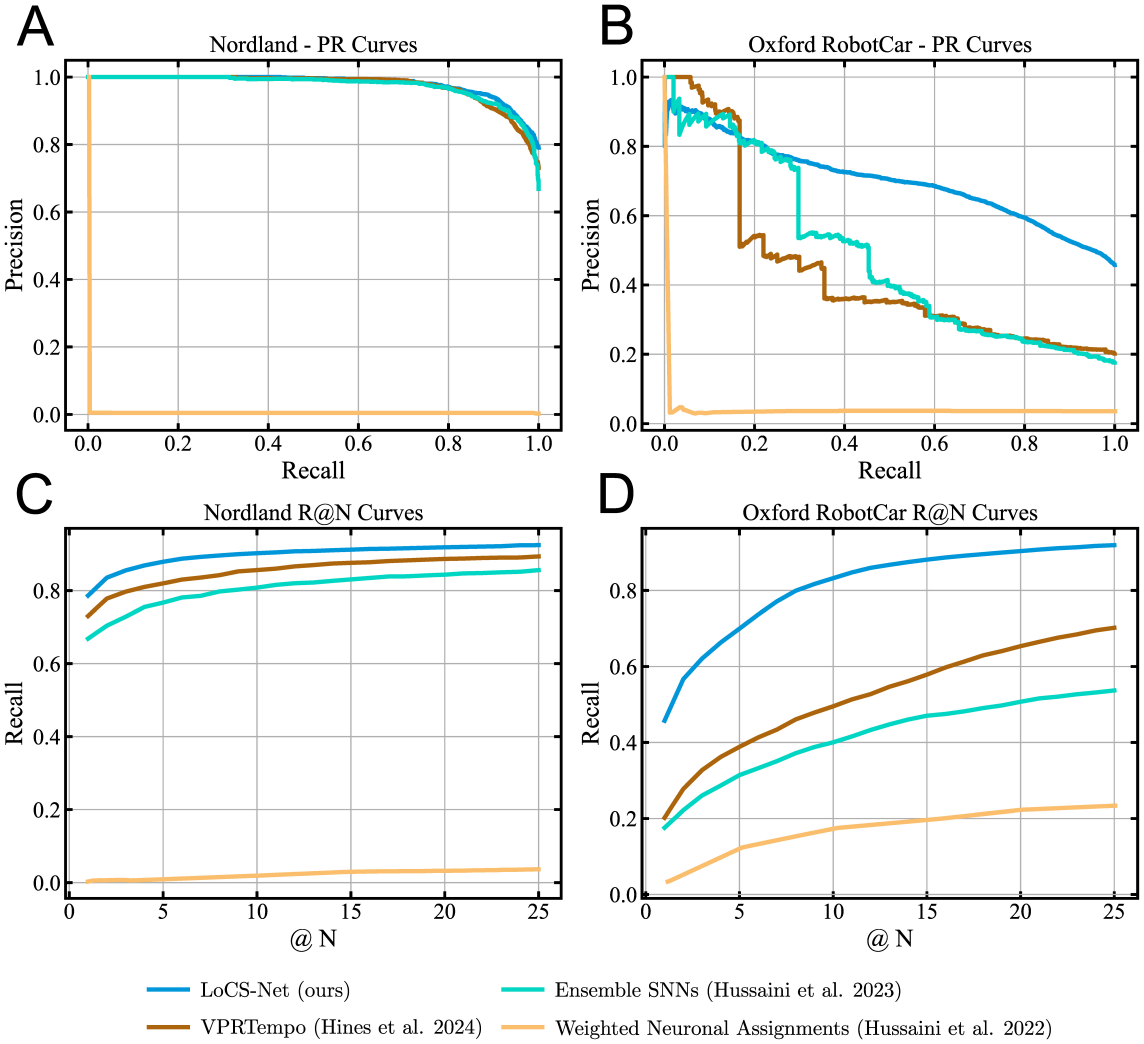
Precision-Recall and Recall @ N curves for the baseline SNN-based VPR methods and LoCS-Net: The blue, brown, cyan, and orange curves correspond to LoCS-Net, VPRTempo (Hines et al., 2024), Ensemble SNNs (Hussaini et al., 2023), and Weighted Neuronal Assignments (Hussaini et al., 2022), respectively. These figures demonstrate that LoCS-Net yields the best SNN-based VPR performance on both datasets. **(A)** PR curves obtained from the experiments on the Nordland dataset. **(B)** PR curves obtained from the experiments on the ORC datasets. **(C)** The R@N curves obtained from the experiments on the Nordland dataset. **(D)** The R@N curves obtained from the experiments on the ORC dataset.

Table 2 and Figure 6 summarize the VPR performance of LoCS-Net along with the reference methods. We observe that LoCS-Net outperformed all the SNN-based methods on both the Nordland (Olid et al., 2018) and ORC dataset (Maddern et al., 2017, 2020) by a large margin (78.6% and 45.7% respectively) in terms of P@100%R. LoCS-Net took much less time to train as reported in Table 2, which highlights LoCS-Net's compatibility for rapid prototyping and real-world deployment. Although it falls short of top-performing ANNs such as MixVPR and Conv-AP, LoCS-Net's strengths lie in energy efficiency and training time. While its GPU-based energy usage (2.545J) sits between that of ANNs like EigenPlaces (1.283J) and AP-GeM (5.376J), deploying LoCS-Net on neuromorphic hardware (Loihi) drastically reduces energy consumption, reaching just 0.032J per inference.

Moreover, Figures 6C, D present the Recall @ N curves obtained from the evaluations of the methods on the Nordland (Olid et al., 2018) and ORC datasets (Maddern et al., 2017, 2020). LoCS-Net consistently yields the best Recall @ N performance compared to SNN methods on both datasets. These results indicate good scalability of the LoCS-Net model across thousands of locations, while maintaining computationally efficient inference, as illustrated by Table 2.

**Figure 7:**
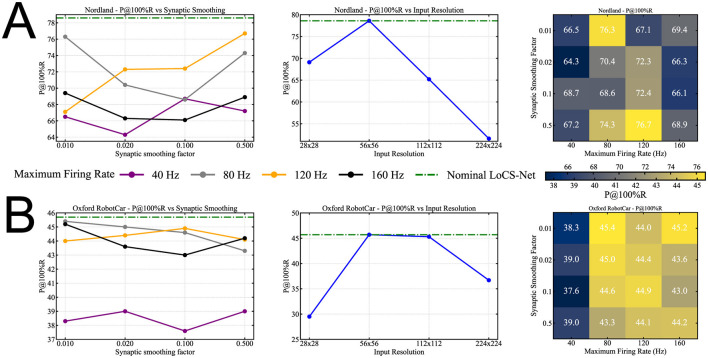
VPR performance in P@100%R sensitivity of LoCS-Net on Nordland **(A)** and ORC **(B)** datasets with respect to synaptic smoothing factor, maximum firing rate, and input layer resolution: The remaining neuronal and training parameters of the nominal LoCS-Net model (see Supplementary Tables S1, S2) are kept the same. Curves in the synaptic smoothing sensitivity plots (on the left) are color-coded, indicating model instances with different maximum firing rates with a nominal input resolution of 56 × 56. Plots in the middle depict the sensitivity with respect to only input resolution. Dashed green curves represent the performance of the nominal LoCS-Net model instance. Synaptic smoothing factor-maximum firing rate tables (on the right) illustrate the same data presented in the plots on the left. These tables contain the P@100%R values in white text while exhibiting the corresponding color codes at the same time. The P@100%R values are obtained by averaging the simulation results of five models, each initiated with a different seed for synaptic weights. The results suggest that LoCS-Net is sensitive to the synaptic smoothing factor, maximum firing rate, and input layer resolution, especially on Nordland data.

We conduct a sensitivity analysis of LoCS-Net with respect to the number of neurons used for signal representation, the maximum firing rate, and the synaptic smoothing factor. We note that the nominal LoCS-Net does not include synaptic filters in order to avoid the additional complexity imposed by temporal dynamics during training, as capturing precise synaptic dynamics is not our primary objective. Figure 7 presents the P@100%R sensitivity analysis of LoCS-Net on the Nordland Figure 7A and ORC Figure 7B datasets, focusing on the synaptic smoothing factor, maximum firing rate, and input layer resolution. All other neuronal and training parameters of the nominal LoCS-Net model remain unchanged. In Figures 7A, B, the synaptic smoothing sensitivity plots on the left use color-coded curves to represent different maximum firing rates for a nominal input resolution of 56 × 56. The middle plots isolate the effect of input resolution on model sensitivity, with dashed green curves showing the performance of the nominal LoCS-Net configuration. On the right, tables summarize the combined influence of the variances in synaptic smoothing factor and maximum firing rate, providing the same information as the left-hand plots. The P@100%R values of Figure 7 are computed as averages across simulations of five models initialized with different seeds per parameter set. The findings highlight that LoCS-Net is sensitive to variations in synaptic smoothing factor, maximum firing rate, and input layer resolution, with sensitivity being particularly evident on the Nordland dataset.

**Figure 8:**
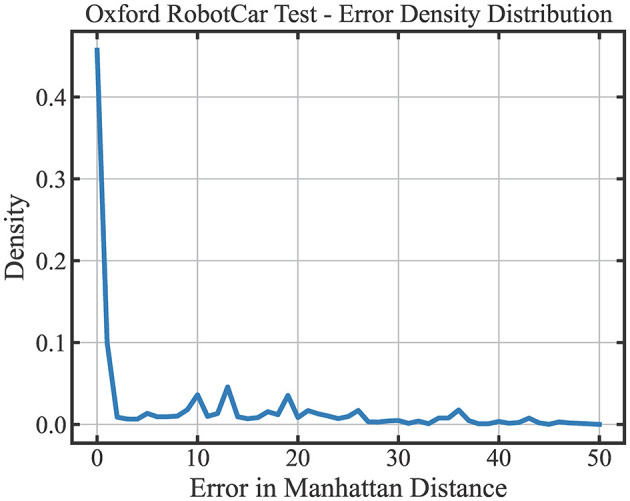
Prediction error distribution of LoCS-Net over the ORC dataset: 55.7% of the place predictions of LoCS-Net are within 1-Manhattan Distance of the ground truth labels. Approximately 40% of the LoCS-Net place predictions fall beyond 5-Manhattan Distance of the ground truth labels.

We further seek to understand the distribution of LoCS-Net's prediction errors on the ORC dataset. We quantify the prediction error in in terms of Manhattan Distance, as illustrated in Figure 8. 55.7% of the place predictions are within 1-Manhattan Distance of the ground truth labels. Yet, approximately 40% of the LoCS-Net place predictions fall beyond 5-Manhattan Distance of the ground truth labels. We did not perform the same analysis for Nordland data as it doesn't utilize a grid-based labeling structure as the Oxford RobotCar data.

### 4.4 Neuromorphic hardware deployment

We deployed the trained LoCS-Net on Kapoho Bay, a USB form that hosts 2 of Intel's neuromorphic Loihi chips (Davies et al., 2021). We utilized NengoLoihi (DeWolf et al., 2020) to deploy LoCS-Net on the Loihi chips. The hardware supports up to 260M trainable synaptic connections with 260k neurons; however, the network structure must be sufficiently tuned to fully utilize the hardware due to its architecture.

After the network parameters are trained, neurons and connections must be distributed between two chips, with 128 neuromorphic cores in each. Each core is designated to handle 1,024 neurons at a time, and the number of core-to-core connections is restricted to about 4,000 synapses due to the limited synapse memory. Therefore, networks with large input/output connections must be partitioned across several cores, and the biases are removed from the convolutional layers to reduce inter-core communication. These strategies have be followed to avoid under-utilization of the cores. As a result, our hardware-deployed network architecture contains fewer trainable parameters with sparse connections due to the above constraints, which may result in a slight decrease in performance. Additionally, as the Kapoho Bay is optimized for mobile deployment and energy efficiency, the device handles spike-timing with 8-bit accuracy, which defines its quantization limit. Training the simulated network without accounting for these hardware specifications might lead to performance drop during on-chip inference. To minimize such discrepancies, the regularization parameter of the training is tuned to adjust the magnitude of the network weights. Here, we note that the hardware limitations mentioned above may be resolved in future versions of neuromorphic chips.

**Figure 9:**
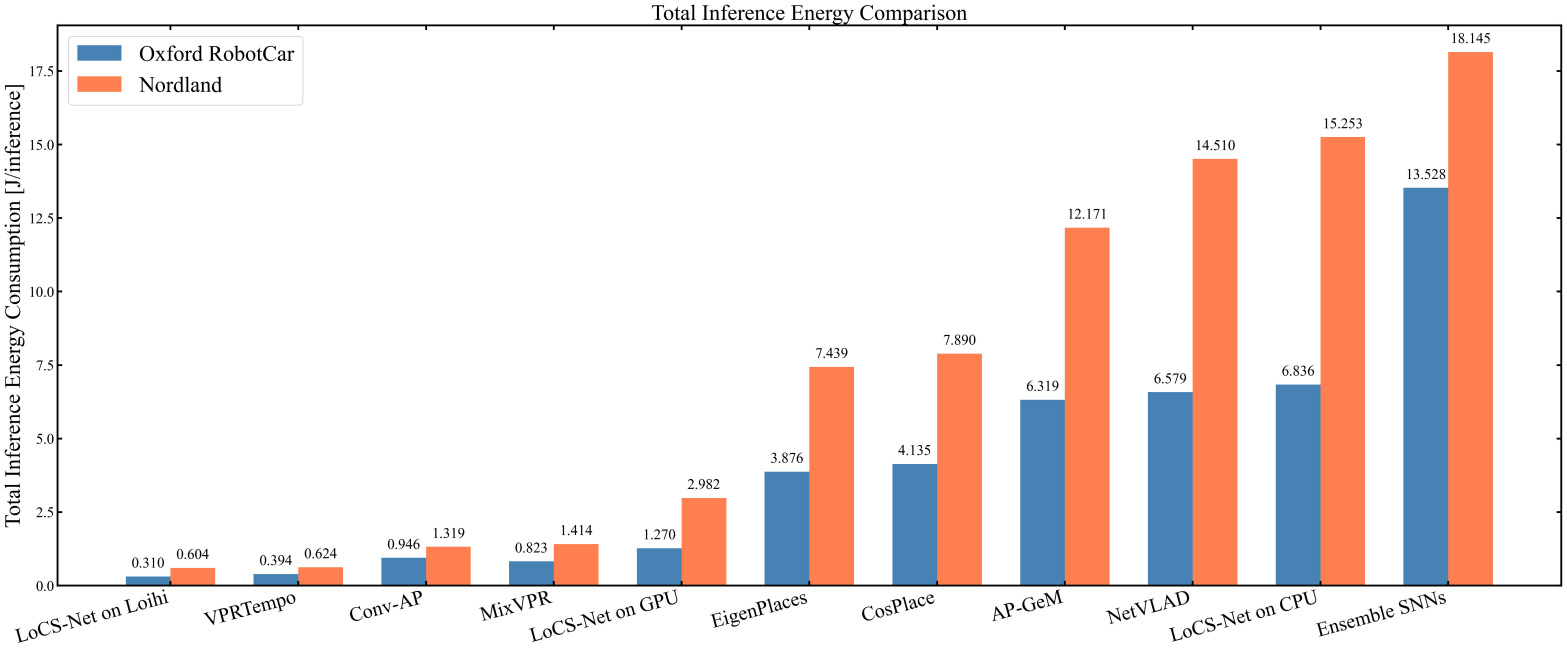
Total energy consumption (in Joules) per inference for different VPR methods: Orange bars represent energy consumption on Nordland data, while blue bars correspond to ORC data. The LoCS-Net model deployed on Loihi achieves the lowest energy consumption per inference, closely followed by its on-GPU SNN competitor, VPRTempo.

To examine the energy-saving benefits of neuromorphic hardware, we measured the average energy consumption per inference of LoCS-Net when deployed on Loihi, Intel NUC7i7BNH (a small-form-factor PC suitable for mobile robotics), and GPU (NVIDIA RTX 3060). We utilized pyJoules (Belgaid et al., 2019) to measure the average energy consumption on the GPU and NUC, while employing a standard off-the-shelf USB tester to observe the power drawn by Kapoho Bay. For each type of hardware hosting LoCS-Net, we first measured the idle power and then the power drawn under load while LoCS-Net was operational. We then subtracted the idle power from the load (or total) power to obtain the closest estimate of LoCS-Net's effective energy consumption, which we list in Table 2. Moreover, we report the total inference energy values in Figure 9.

## 5 Discussion

We observe a noticeable performance drop of the on-chip LoCS-Net, while achieving at least an order of magnitude improvement in energy efficiency on both datasets compared to CPU and GPU deployments. We believe that the gradient mismatch between the LIF neurons (Burkitt, 2006) and their rate approximations (Hunsberger and Eliasmith, 2015) significantly contribute to the reduced performance of LoCS-Net in this case, as also mentioned by Che et al. (2022). LoCS-Net outperforms all SNN-based methods on both datasets in terms of area-under-the-precision-recall curves, while demonstrating the second fastest inference as shown in Table 2. VPRTempo turns out to be the fastest (in terms of inference time) and the most energy-efficient simulated SNN, coming close after on-chip LoCS-Net in terms of energy consumption per inference. However, it fails to exhibit robustness against dynamic scenes, noise, variance in viewpoint, and lighting conditions in the ORC dataset.

We observe relatively poor performance of our method on the ORC dataset (Maddern et al., 2017, 2020). In addition to ANN-to-SNN conversion losses, we hypothesize that the more dynamic scene content of the ORC images (Maddern et al., 2017, 2020) and the substantial noise levels in a significant portion of the test ORC images impede better VPR performance of LoCS-Net.

As reported in Table 2, LoCS-Net consumes 0.06 J per inference when processing the Nordland images, approximately 1/40th of the energy consumed by the GPU and about 1/220th of the energy consumed by the CPU of the NUC. Similarly, Loihi chips demonstrate the greatest energy efficiency (0.032 J/inference vs. 5.871 J/inference on NUC and 1.095 J/inference on GPU) by a large margin when processing the ORC images. We consistently observe the total energy consumption of neuromorphic chips does not scale intuitively with respect to the size of the neural network in terms of the number of trainable parameters. Instead, the inference energy cost appears to be more related to the communication time between the integrated CPU and the Loihi chip. This includes the time required to generate and to send the spike signals through the input layer of LoCS-Net using the integrated CPU within the Loihi device, and to decode the output signal back to numerical data. This observation suggests that a significant restriction of energy efficient neuromorphic computation involves data conversion during encoding and decoding, which must be managed by traditional CPU architecture. The communication bottleneck also affects the total inference time. Due to the communication delay, there is a challenge in optimally including the spike-conversion stage in-between the sensing and input neurons, as well as between the output layer and the actuator. Unfortunately, this spike-conversion step scales linearly with the data size and the resolution we aim to represent. However, testing the proposed method on alternative neuromorphic hardware designs (Hazan and Ezra Tsur, 2022; Halaly and Ezra Tsur, 2023) might offer even greater power efficiency and yield faster inference times.

The overall performance of the SOTA ANN methods proved superior to that of their SNN competitors in terms of precision at 100% recall on both the Nordland and ORC datasets. As shown in Table 2, while these SOTA ANN techniques outperform their SNN competitors, they also require significantly longer time to generate descriptors (loosely corresponding to training time) and to compute reference-query matches (corresponding to inference time) compared to LoCS-Net. On-chip LoCS-Net remains the most energy-efficient VPR method, with the fastest training time by a large margin.

We must also note that the SOTA ANN approaches included in our comparison studies use pre-trained networks (e.g., ResNet50 and ResNet101 backbones), which are subsequently fine-tuned for the VPR tasks. In contrast, LoCS-Net is trained solely on data from the navigation domain of interest. In this sense, comparing our network to these SOTA ANN techniques may not be entirely fair, as they benefit from cumulative training over a much larger dataset. We would like to emphasize that our work focuses on the SNN domain, and LoCS-Net significantly advances the state of the art in SNN-based VPR techniques.

**Figure 10:**
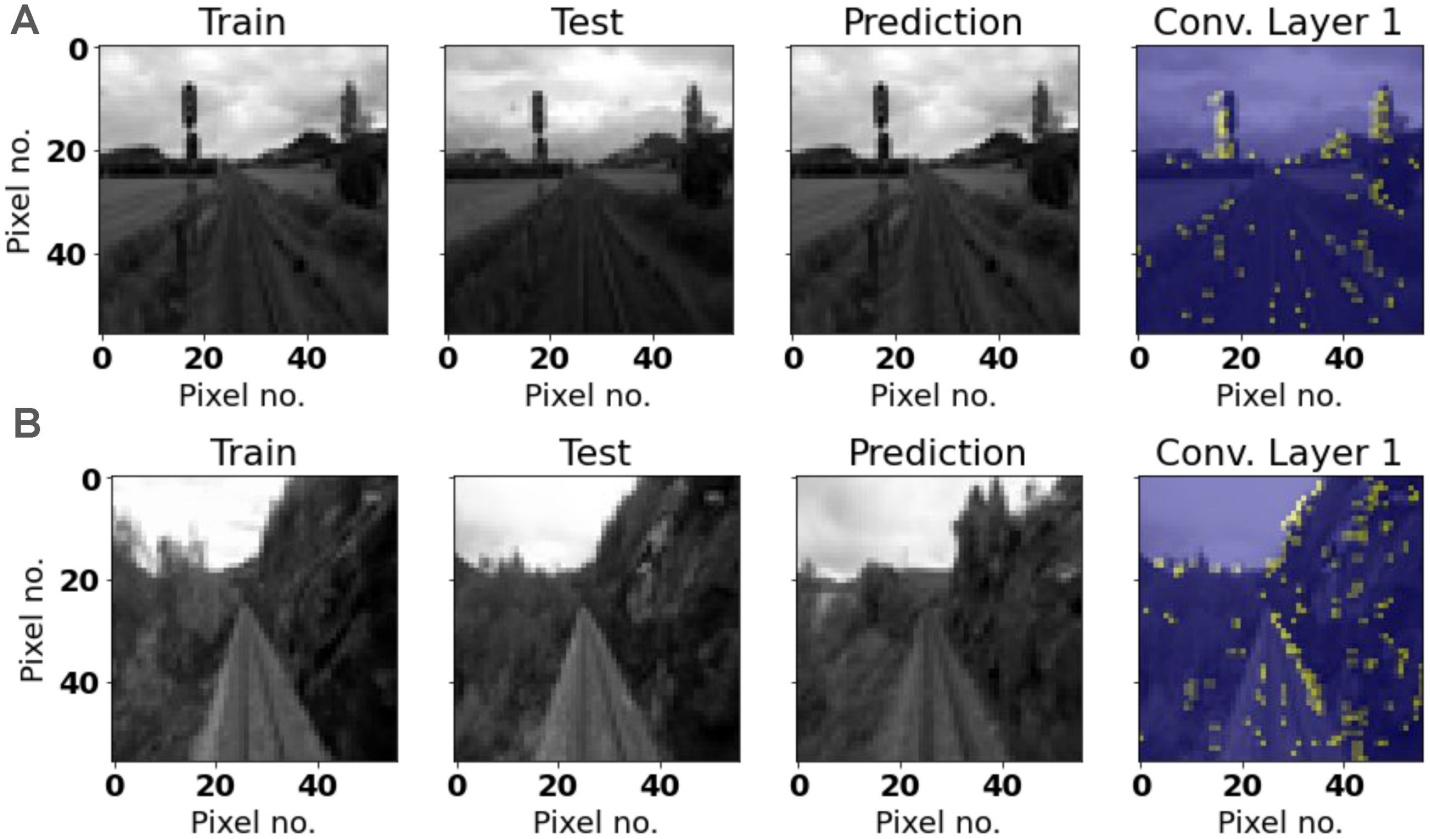
Representative samples of Nordland training and test images: Yellow regions represent the associated active neurons. **(A)** An instance of correct prediction. Representative convolutional layer activation appears to represent features in the input image. **(B)** An instance of incorrect prediction. Although the activation of the convolution layer appears to represent features of the image, the prediction made is for a similar, but incorrect class label.

**Figure 11:**
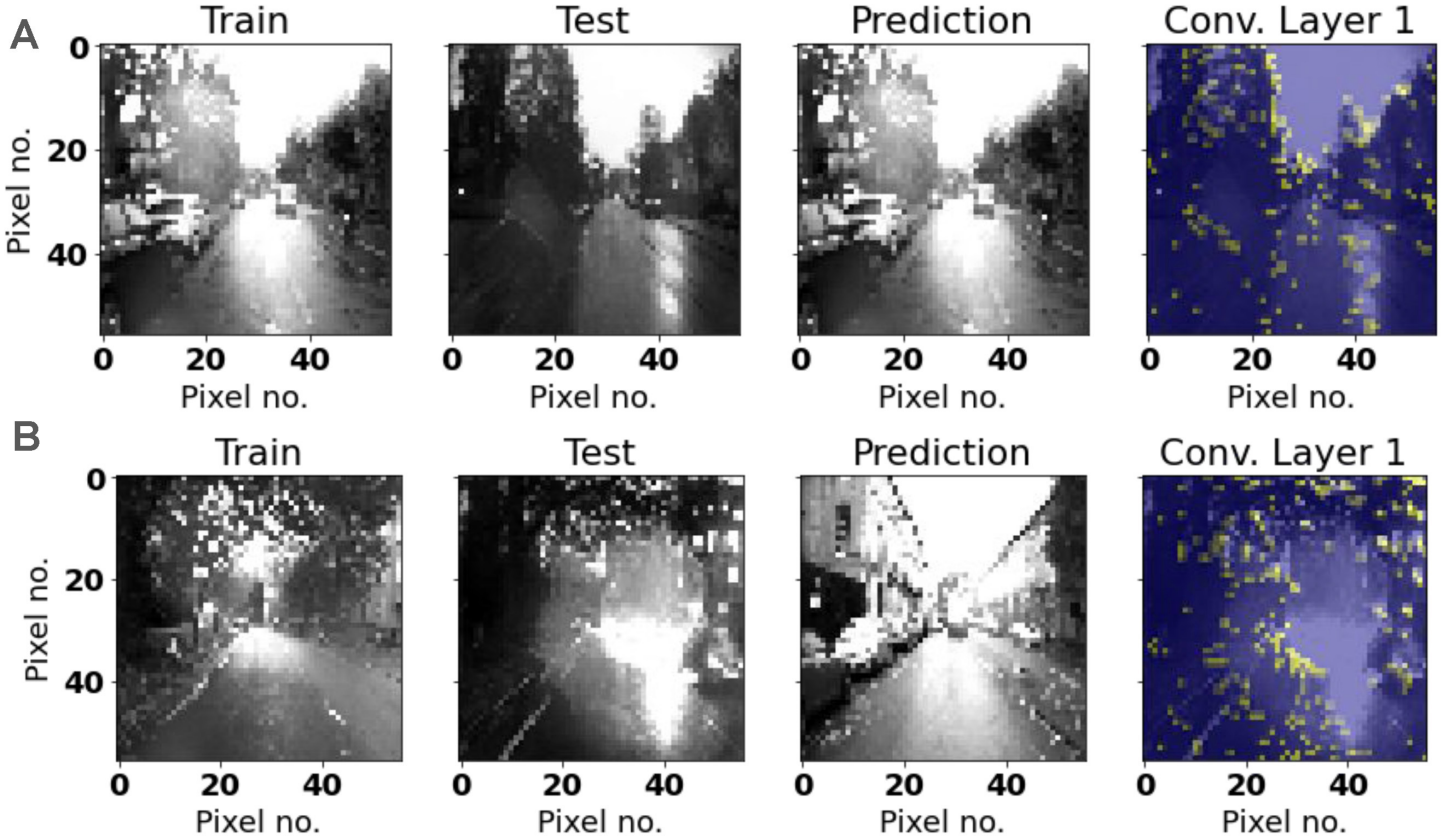
Representative samples of Oxford Robot Car training and test images: Yellow regions represent the associated active neurons. **(A)** An instance of correct prediction. Representative convolutional layer activation appears to represent features in the input image. **(B)** An instance of incorrect prediction. A possible water droplet on the lens causes significant image distortion that results in incorrect place prediction.

We may further analyze the performance of LoCS-Net by investigating the spiking activity in the convolutional layers of the model. Figures 10, 11 depict representative samples of Nordland and ORC training and test images. Compared to the ORC images, Nordland test and training instances are much more visually aligned. ORC test images, on the other hand, are extremely challenging due to intense variance in lighting, appearance, viewpoint, and noise. Some of these test instances are impossible to recognize by a human observer. We believe that these characteristics of the ORC data significantly contribute to LoCS-Net's reduced performance on this dataset.

We examined the activities generated by a set of randomly chosen images that were either correctly or incorrectly labeled by LoCS-Net. The images shown in this section are representative samples for both mislabeled and correctly labeled images from the Nordland and Oxford RobotCar datasets. We observed similar spiking activity patterns in all of the images we randomly picked as in those demonstrated in Figures 10, 11.

Figures 10, 11 depict the spiking unit activations of the first convolutional layer in the model, when presented images from the Nordland and ORC datasets. In both figures, the correct predictions are associated with spiking activity that is clustered over large features in the input image that could potentially help to distinguish the input image from others. When we examine the spiking activities generated by the images that are mislabeled by LoCS-Net, we observe matching spiking patterns with the activities generated by the training image for the correct class. This implies that LoCS-Net struggles to distinguish images marginally different from each other.

## 6 Conclusion

In this work, we formulate visual place recognition as a classification problem and develop LoCS-Net, a convolutional SNN to solve VPR tasks with challenging real-world datasets. Our approach leverages ANN-to-SNN conversion and back-propagation for tractable training, by using rate-based approximations of leaky integrate-and-fire (LIF) neurons. The proposed method substantially surpasses existing state-of-the-art SNNs on challenging datasets such as Nordland and ORC, achieving 78.6% precision at 100% recall on the Nordland dataset (compared to the current SOTA's 73.0%) and 45.7% on the Oxford RobotCar dataset (compared to the current SOTA's 20.2%). Our approach simplifies the training pipeline, delivering the fastest training time and the second fastest inference time among SOTA SNNs for VPR. Hardware-in-the-loop evaluations using Intel's neuromorphic USB device, Kapoho Bay, demonstrate that our on-chip spiking models for VPR-trained through the ANN-to-SNN conversion strategy-continue to outperform their SNN counterparts, despite a slight performance drop when transitioning from off-chip to on-chip, while still offering significant energy savings. These results emphasize the LoCS-Net's exceptional rapid prototyping and deployment capabilities, marking a significant advance toward more widespread adoption of SNN-based solutions in real-world robotics.

The SOTA ANN methods over-shadowed their SNN counterparts in terms of precision at 100% recall on both the Nordland and ORC datasets. However, as detailed in Table 2, these ANN techniques come with notable trade-offs, requiring longer times for descriptor generation and inference relative to LoCS-Net. Among the evaluated methods, the on-chip implementation of LoCS-Net stands out as the most energy-efficient VPR solution, achieving the shortest training time by a considerable margin.

We would like to emphasize that this manuscript proposes LoCS-Net as a environment-specific VPR solution. In that sense, providing a long-term general spatial memory as a global VPR solution is beyond the capabilities of the current work. In addition, LoCS-Net's performance is sensitive to the definition of places, which in turn may require the implementation of domain-specific discretization techniques to maximize the performance of LoCS-Net over different navigation environments. As discussed in Section 4.3, LoCS-Net doesn't perform as well over the Oxford RobotCar (Maddern et al., 2017, 2020) data as compared with the Nordland (Olid et al., 2018) dataset. This might be due to the LIF neuron approximation errors as well as the significantly varying lighting and road conditions of the ORC traverses (Maddern et al., 2017, 2020), which suggests the lack of robustness to such dynamic scenes. Nevertheless, we emprically show that LoCS-Net is much better than its SNN competitiors at handling such challenges.

## Data Availability

The computational model and data preparation scripts generated for this study can be found in the FigShare repository at https://figshare.com/s/c159a8680a261ced28b2. This includes all necessary code to reproduce the results presented in this paper. Please direct further inquiries and questions about the model implementation to the corresponding authors.
