# Supplementary material for: LoCS-Net: Localizing convolutional spiking neural network for fast visual place recognition
Source: Front Neurorobot. 2025 Jan 29;18:1490267. doi: 10.3389/fnbot.2024.1490267 (PMC11813887; doi:10.3389/fnbot.2024.1490267)
Supplement: Supplementary file 1 [file Data_Sheet_1.pdf]

Supplementary Material

Table S1. Neuronal, training, and testing hyer-parameters of LoCS-Net on the Nordland (22) dataset.

Hyper-parameter	Value
Place sample frequency	8
Input Resolution	56x56
Training Epoch Number	15
Batch Size	96
Inference Simulation Time Step	100
Learning Rate	0.001
Output Layer Read-out Time Constant	0.1
LIF Neuron Amplitude	0.01
Maximum Spiking Rate	100
Seed Number	0
Place Nunmber	3,072
Number of Trainable Parameters	56,873,856
LIF Response Curve Intercepts	0
Convolutional Layers' Kernel Size	3
Convolutional Layers' Padding	0
1st Convolutional Layer's Stride	1
2nd and 3rd Convolutional Layers' Stride	2
Convolutional Layer #1, #2, and #3's Kernel Number	32, 64, and 128

Table S2. Neuronal, training, and testing hyer-parameters of LoCS-Net on the ORC (23,24) dataset.

Hyper-parameter	Value
Grid Resolution	50x50
Input Resolution	56x56
Training Epoch Number	15
Batch Size	110
Inference Simulation Time Step	50
Learning Rate	0.001
Output Layer Read-out Time Constant	0.1
LIF Neuron Amplitude	0.005
Maximum Spiking Rate	Uniform Distribution: $\rho_{max}{\sim}U(80, 160)$
Seed Number	0
Place Nunmber	185
Number of Trainable Parameters	3,657,785
LIF Response Curve Intercepts	0
Convolutional Layers' Kernel Size	3
Convolutional Layers' Padding	0
1st Convolutional Layer's Stride	1
2nd and 3rd Convolutional Layers' Stride	2
Convolutional Layer #1, #2, and #3's Kernel Number	32, 64, and 128
